# Organophosphorus Poisoning among Patients Admitted to the Intensive Care Unit of Department of Internal Medicine in a Tertiary Care Centre: A Descriptive Cross-sectional Study

**DOI:** 10.31729/jnma.7823

**Published:** 2022-09-30

**Authors:** Suzit Bhusal, Rupa Bhandari, Sujata Dahal, Aliska Niroula, Krity Basnet, Ashlesha Chaudhary, Subash Pant

**Affiliations:** 1 Kathmandu Medical College and Teaching Hospital, Sinamangal, Kathmandu, Nepal; 2 Jibjibe Rural Hospital, Jibjibe, Rasuwa, Nepal; 3 Nepal Medical College and Teaching Hospital, Jorpati, Kathmandu, Nepal; 4 Department of Internal Medicine, Kathmandu Medical College and Teaching Hospital, Sinamangal, Kathmandu, Nepal

**Keywords:** *Nepal*, *organophosphorus poisoning*, *prevalence*

## Abstract

**Introduction:**

Organophosphates are potent cholinesterase inhibitors that when ingested in excessive amounts can be fatal. Organophosphorus poisoning has become an important clinical problem with increased mortality in the country from accidental or intentional ingestion of, or exposure to the pesticide. This study aimed to find out the prevalence of organophosphorus poisoning among patients admitted to the Intensive Care Unit of Department of Internal Medicine in a tertiary care centre.

**Methods:**

A descriptive cross-sectional study was conducted among patients admitted to the Intensive Care Unit of the Department of Internal Medicine in a tertiary care centre after receiving ethical approval from the Institutional Review Committee (Reference number: 2003202205). The study was conducted between 1 February 2021 and 1 February 2022 using hospital records. Convenience sampling was done among the patients who met the eligibility criteria. The diagnosis of organophosphorus poisoning was made based on the patient's history, clinical examination and the measurement of serum acetylcholinesterase levels. Point estimate and 95% Confidence Interval were calculated.

**Results:**

Among 1108 patients admitted, organophosphorus poisoning was seen in 50 (4.51%) (3.295.73, 95% Confidence Interval).

**Conclusions:**

Our study found that the prevalence of organophosphorus poisoning was lower when compared to similar studies done in similar settings.

## INTRODUCTION

Organophosphates are potent cholinesterase inhibitors capable of causing severe cholinergic toxicity following accidental or intentional cutaneous exposure, inhalation, or ingestion.^12^ Respiratory failure and lung injury remain the primary cause of mortality among patients with organophosphorus (OP) poisoning. However, variability exists in the clinical symptoms and signs depending on the nature of the poison, amount consumed, severity, duration between exposure, and presentation in the hospital.^3^

Poisoning with organophosphorus pesticides has become a major clinical and public health problem with increasing case fatality rates across much of rural Asia.^1,4^ Studies have reported pesticide poisoning to account for more than 60% of the estimated 500,000 deaths from self-harm.^1,5^ However, limited information is available on the status and prevalence of different pesticide poisoning in Nepal.

This study aimed to find out the prevalence of organophosphorus poisoning among patients admitted to the Intensive Care Unit (ICU) of Department of Internal Medicine in a tertiary care centre.

## METHODS

A descriptive cross-sectional study was conducted among patients admitted to the ICU of the Department of Internal Medicine of a Kathmandu Medical College and Teaching Hospital (KMCTH) after receiving ethical approval from the Institutional Review Committee (Reference number: 2003202205). The study was conducted between 1 February 2021 and 1 February 2022. All the patients who were diagnosed with organophosphorus poisoning in the department of Medicine department of KMCTH within the study period were included in the study. However, the patients who had mixed ingestion of poisons were excluded from the study. Convenience sampling was done and the sample size was calculated using the following formula:


$$\begin{array}{ll} \text{n} & ={\text{Z}}^{2}\times \frac{\text{p}\times \text{q}}{{\text{e}}^{\text{2}}}\\ & =1.96^{2}\times \frac{0.5\times 0.5}{0.03^{2}}\\ & =1068 \end{array}$$

Where,

n= minimum required sample size
Z= 1.96 at 95% Confidence Interval (CI)
p= prevalence taken as 50% for maximum sample size calculation
q= 1-p
e= margin of error, 3%

The minimum sample size calculated was 1068. However, a sample size of 1108 was taken for the study. The diagnosis of OP poisoning was made primarily on clinical grounds. Detailed history, clinical findings and examination findings were studied. An assay for plasma acetylcholinesterase activity was also performed.^6^ Data regarding sociodemographic factors, amount of poison ingested, arrival time since exposure, concurrent alcohol intake, clinical features and outcome of the patients were also collected from the hospital records of the ICU of the Department of Internal Medicine of KMCTH.

Data were collected and then entered and analysed in Microsoft Excel. Point estimate and 95% CI were calculated.

## RESULTS

**Figure 1:**
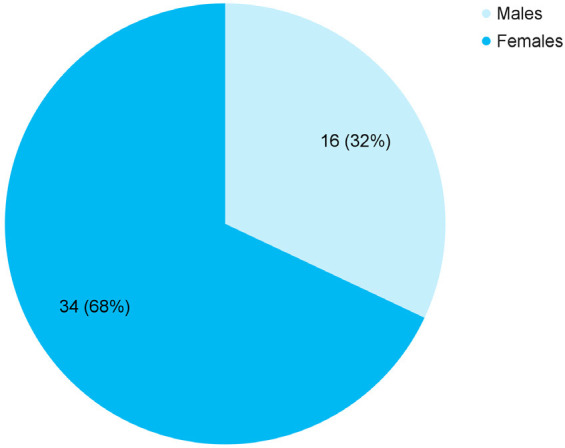
Gender-wise distribution of the patients with OP poisoning (n= 50).

Among 1108 patients admitted, prevalence of OP poisoning was seen in 50 (4.51%) (3.29-5.73, 95% CI). The mean age of the patients was 34.22±16.14 years and 34 (68%) were females (Figure 1)

**Figure 2:**
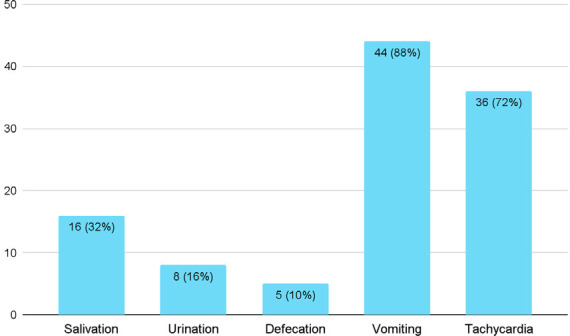
Clinical features at presentation (n= 50)

Forty-four (88%) of the patients with OP poisoning could provide an estimate of the amount of poison ingested which was a median value of 37.5 ml. Thirty-five (70%) of the patients consumed ≤100 ml, 2 (50%) consumed 100-200 ml, 5 (50%) consumed 300-400 ml whereas 1 (2%) consumed >4000 ml of OP. The mean time of arrival of the patients after ingestion of the poison was 2.76±1.74 hours ranging from 30 minutes to 8 hours. Regarding their history, 11 (22%) patients had a history of concurrent alcohol intake and 1 (2%) had a history of substance abuse. The most common clinical feature of the patients was vomiting which was seen in 44 (88%) patients followed by tachycardia which was seen in 36 (72%) patients (Figure 2).

The median acetylcholinesterase level of these patients was 738.50 U/l. Fifteen (30%) of the patients had serum acetylcholinesterase levels <500, 11 (22%) had levels between 500-1000, 5 (10%) had levels between 10002000, 6 (12%) had levels between 2000-4000, 5 (10%) levels between 4000-8000, 3 (6%) levels between 800010000 and 1 (2%) levels >10000. The median maximum dose of atropine provided to these patients was 15 ml/hr. The mean of total duration of the treatment was 13.20±7.88 days. The details of the management of these patients are tabulated below (Table 1).

**Table 1:** Details on management of the patients (n= 50).

Parameters	n (%)
Gastric lavage	30 (60)
Oxygen requirement	25 (50)
Requirement of ventilator support	15 (30)
Mortality	4 (8)

## DISCUSSION

OP poisoning has become an important issue in the context of Nepal, with increasing prevalence and mortality across the country. Our study gave the prevalence of 4.15% OP poisoning cases among patients admitted to the ICU in a tertiary care centre in one year.

A descriptive cross-sectional study conducted in Chitwan among 439 acute pesticide poisoning cases gave the prevalence of OP poisoning to be 37.3% which was higher than our study.^7^ Other studies in Nepal have shown a similar prevalence of 51.95%,^8^ 42%,^9^ 32.31%,^10^ and 67%.^11^ Our study showed female predominance which was similar to other studies.^7-9^ However, the time elapsed between exposure and hospital arrival ranged from 10 minutes to 22 hours which was different from our study. The mortality rate of this study was 3.8% which was lower than ours.

The mean age years of the patients in our study was 34.22±16.14 years. Other studies have also reported OP poisoning to be more common among the younger population.^7,12^ The vulnerability of this population could be reflected by this, particularly during the time of life's major stressors to stress, maladjustment, and immature psychological coping mechanisms.^13^

The average length of stay and treatment in the hospital was 13.20±7.88 days which was higher when compared to other similar studies.^14^ The requirement of respiratory support in the study was seen in 30% of the patients and mortality in 8% which was higher than in similar studies.^7,14,15^ This could reflect the need for better health facilities and policies targetting early diagnosis and management of poisoning in Nepal and possible prevention of further poisoning cases in the country for a reduction in the prevalence.

This study had some limitations. A larger sample size and study involving multiple centres could increase the generalizability of the study. Also, the association between the variables such as the age, gender, amount of the poison ingested, arrival time since exposure, treatment received and the outcome could not be made in this study design. Risk factors could not be made out as well. Higher studies are recommended. Lastly, the nature of the exposure to the poison that is whether it was accidental or suicidal was not studied.

## CONCLUSIONS

Our study found that the prevalence of OP poisoning was lower when compared to similar studies done in similar settings. Frequent reports of the poisoning among younger age groups and females could reflect their vulnerability. However, higher studies are warranted for the study of the possible factors associated with increased risk of poisoning which could have some impact and implications in lowering the prevalence of OP poisoning.
